# Exploring the Zoonotic Risk of *Bartonella henselae*: A Serological and Molecular Investigation of Veterinary Personnel and Companion Cats in South Korea

**DOI:** 10.1155/tbed/2468636

**Published:** 2025-10-24

**Authors:** Keon Kim, Minseo Kim, Byung-Yeol Lee, Chang Hyeon Choi, Hyun Jin Kim, Woong Bin Ro, Kwang Jun Lee, Sung-Hak Kim, Chang-Min Lee

**Affiliations:** ^1^Department of Veterinary Internal Medicine, College of Veterinary Medicine and BK21 Four Program, Chonnam National University, Gwangju 61186, Republic of Korea; ^2^Animal Molecular Biochemistry Laboratory, Department of Animal Science, College of Agriculture and Life Sciences, Chonnam National University, Gwangju 61186, Republic of Korea; ^3^Division of Zoonotic and Vector Borne Disease Research, National Institute of Health, Korea Disease Control and Prevention Agency, Cheongju, Republic of Korea

**Keywords:** Bartonella, feline, Korea, people, prevalence, veterinary personnel, zoonosis

## Abstract

*Bartonella* species are known as candidates for zoonotic transmission, and cats serve as the main reservoir for *Bartonella henselae*, the primary causative agent of cat scratch disease (CSD). However, research on the transmission of bartonellosis to humans remains very limited. In East Asia, there is a lack of comprehensive studies regarding the prevalence of *B. henselae* infection and its associated risk factors among companion cats, their owners, and individuals working in veterinary professions. This study aimed to investigate both the molecular and seroprevalence of *B. henselae* in veterinary personnel and companion cats in South Korea, along with a questionnaire-based analysis of transmission risk factors. Blood and saliva samples were collected from humans, whereas blood, saliva, claw, and fecal samples were obtained from cats. Seroprevalence and molecular prevalence were measured for all these samples. Additionally, participants were required to complete an epidemiological information questionnaire related to CSD. The study enrolled 300 veterinary professionals and 126 companion cats owned by them. The serum IgG prevalence in humans was 64.6% (190/294), whereas, in cats, it was 5.5% (6/108). The molecular prevalence in human blood and saliva was 3% (9/298) and 1.7% (5/298), respectively. In cats, it was 10.1% (12/119) for blood, 0% (0/123) for saliva, 1.7% (2/119) for nails, and 4.5% (5/112) for feces. Phylogenetic analysis of the PCR-positive samples confirmed that all of them were *B. henselae*. This study demonstrates that Bartonella species are widespread among veterinary professionals in South Korea, highlighting their significance as zoonotic pathogens. Given the potential for indirect transmission from cats, enhancing awareness of Bartonella exposure risk among veterinary personnel is warranted, along with emphasizing preventive education for cat owners, including strict ectoparasite control.

## 1. Introduction

*Bartonella* spp. are fastidious and hemotropic bacteria with a Gram negative nature that are primarily spread through vectors [1]. Domestic cats serve as the main reservoir for *Bartonella henselae*, the primary causative agent of cat scratch disease (CSD) [2]. Clinical signs resulting from *B. henselae* infection are marked by the development of papules and pustules at the site of the scratch, along with swollen lymph nodes within 1–3 weeks. Additionally, individuals frequently experience mild fever, malaise, and aching [1]. According to recent studies, the genus *Bartonella* comprises more than 45 species and 20 *Candidatus* [3–5], among which at least 13 have been identified as causative agents of zoonotic infections [6, 7]. While not conclusively proven, it is reasonable to regard all *Bartonella* species as potential candidates for zoonotic transmission.

The clinical spectrum of infection in cats has not been thoroughly explored, but naturally infected cats mostly appear to carry bacteria without showing signs of illness [2, 8, 9]. However, severe cases involving osteomyelitis, endocarditis, and myocarditis have occasionally been reported [10–14]. Humans may acquire infection indirectly through scratches, bites, or licking from an infected cat. Direct transmission may also occur via flea bites or through contact of contaminated hands—exposed to flea feces—with open wounds or mucous membranes such as the eyes [15]. The primary mode of transmission of *B. henselae* to humans is through skin injuries caused primarily by cat scratches [1]. More recently, other potential vectors, such as ticks and biting flies, have been identified as carriers of *Bartonella* DNA, including *B. henselae* [16, 17]. While the specific role of ticks in *Bartonella* transmission requires further investigation, several recent studies have reported a high prevalence of *Bartonella* spp. infection in ticks from various parts of the world [9, 18, 19]. Additionally, exposure to ticks has already been identified as a risk factor associated with CSD in humans [20].

Recently, due to global warming, a noticeable increase in temperature has occurred. An increase in temperature is correlated with an increase in the incidence of vector-borne diseases [21]. Moreover, the highest infection rates are observed in temperate regions, where conditions are particularly conducive to the proliferation of *Ctenocephalides felis*. Such climate changes suggest a potential increase in the prevalence of bartonellosis as a zoonotic disease. In fact, the high incidence of Bartonella infection has been confirmed by various studies. Approximately 12,500 cases of human bartonellosis are reported per year in the United States [22]. Furthermore, in various European and Asian regions, a substantial seroprevalence, ranging from 8.7% to 19.6%, has been reported even among healthy adults [23–25]. In South Korea, the seroprevalence rate among healthy adults was found to be approximately 15.0% [26]. However, in South Korea, research on bartonellosis has been conducted primarily in the form of case reports in humans [27], and, to date, only a single report published in 2009 has identified the prevalence of *B. henselae* and *B. clarridgeiae* in dogs and cats serving as reservoirs. However, the 2009 report evaluated only the molecular prevalence in cats using a nested PCR method, without assessing the prevalence among pet owners or the analysis of epidemiological risk associated with human-to-cat transmission [28]. Recently, there has been a rapid increase in the companion animal-owning population in South Korea. According to the most recent nationwide survey conducted at the end of 2022, 25.7% of all households were reported to be raising companion animals [29]. This trend has led to more frequent close contact between humans and companion animals, raising concerns about the potential transmission of zoonotic diseases, particularly bartonellosis. Therefore, updated research is needed to systematically assess the current status and risk factors associated with Bartonella infection between companion animals and humans in South Korea.

The considerable geographical variability in the prevalence of *Bartonella* species infections among humans and cats, along with the potentially widespread distribution of risk factors, highlights the need for local assessments of these parameters within each country or region to guide control and monitoring efforts [30]. To date, data regarding the prevalence and transmission risk factors of *B. henselae* infection in cats in East Asia, including South Korea, remain limited. Furthermore, as a zoonotic pathogen, *B. henselae* may pose a particular risk to veterinary personnel, who are frequently exposed to cats. Therefore, the aim of this study was to assess the serological and molecular prevalence of *B. henselae* among clinically healthy companion cats, their owners, and veterinary personnel and to investigate potential risk factors associated with transmission.

## 2. Materials and Methods

### 2.1. Grouping

This study recruited a total of 300 human participants from veterinary clinics and referral animal hospitals distributed nationwide across South Korea. The participants were healthy personnel affiliated with the veterinary hospitals, including veterinarians, technicians, managers, and pet groomers, and were classified as the human study group (*n* = 300). Among them, those who owned cats were retrospectively identified and assigned to the cat owner group (*n* = 126). The healthy cats owned by this group were also enrolled in the study and classified as the feline study group (*n* = 126).

### 2.2. Data and Sample Collection

#### 2.2.1. Questionnaire

Consented participants were asked to complete a questionnaire regarding epidemiological information. The veterinary personnel who did not raise companion cats were only required to answer the “staff questions,” while who raised cats were additionally required to answer the “owner questions.” The staff questions consisted of questions on gender, age, history of overseas travel/business trips, experience with 21 representative clinical symptoms known to be associated with CSD, history of arthropod exposure, history of bites or scratches from companion animals or stray animals, animal hospital size, and daily exposure time to companion animals in the workplace. The owner questions included additional questions such as how long they had lived with their companion cat, the amount of time spent exposed to the cat in the same room, and the duration of direct contact with the companion cat per day. To proceed smoothly with the work, all questionnaires were distributed by the four trained interviewers, and informed consent was obtained from all participants prior to participation in the interview. The original questionnaire items are available in Supporting Information File 1.

#### 2.2.2. Human Samples

Blood and saliva samples were collected from a total of 300 humans. Blood samples were collected for serum biochemical analysis and PCR testing, whereas saliva samples were collected solely for PCR testing. Approximately 6 mL of blood (3 mL in EDTA and 3 mL in a serum separator tube) was collected at enrollment. EDTA tubes were mixed immediately with a blood agitator. The serum separator tubes were left in a standing position for 20–30 min before being centrifuged at 4000 rpm for 15 min. All the samples were stored at −20°C until further examination. Saliva sampling was conducted via a cotton swab moistened with saline solution. After the mouth was lightly rinsed, the swab was gently rubbed against the sublingual area of the tongue for approximately 30–60 s. Samples compromised by handling, transport, or collection deficiencies were excluded from analysis.

#### 2.2.3. Cat Samples

Samples, including blood, saliva, nails, and feces, were collected from a total of 126 cats. Blood samples were collected for both serological and PCR analysis, whereas saliva, nail, and fecal samples were collected exclusively for PCR testing. Approximately 3 mL of blood was collected (1 mL in an EDTA tube and 2 mL in a serum separator tube). Blood samples were processed and stored similarly to those of humans. For saliva sampling, a sterile swab was adequately moistened under the tongue and stored at −20°C. Nail sampling included the use of a sterile swab moistened with saline solution to sample the front and rear nails on both sides. Fecal samples, collected voluntarily or from the rectum, were approximately 2 g each and stored at −20°C. All samples unsuitable due to handling or collection issues were excluded.

### 2.3. IFA Serological Testing

Serum was obtained by centrifuging blood from cats and humans in serum separator tubes (4000 rpm for 15 min). The mixture was subsequently diluted with the diluent in the components of the *B. henselae* feline IFA IgG Kit (Fuller Laboratories, USA) and *Bartonella* IFA IgM, IgG test kit (Focus Diagnostics, France) for human samples. For IgG detection, serum was diluted at a ratio of 1:64, and for IgM detection, serum was diluted at a ratio of 1:20. These dilutions were performed according to the protocols provided with the respective commercial IFA kits used, and no additional serial dilutions were performed beyond these initial dilutions. A total of 20 mL of diluted serum and positive and negative control samples was dispensed onto slides coated with Vero cells infected with *B. henselae*. The bacterial strain used was obtained from the Korea Disease Control and Prevention Agency (KDCA). After the samples were incubated in a humid incubator for 90 min, they were washed with PBS for 10 min. After washing, the samples were soaked in distilled water and air-dried. A total of 20 µL of feline or human IgG–FITC-conjugated antibody was added, and the mixture was reacted at 37°C for 30 min. After the samples were washed with PBS twice for 10 min, they were air dried. After the samples were fixed on the slides, they were compared with the control group at 400 × magnification via a fluorescence microscope to determine positivity according to the degree of fluorescence (cutoff value = 1:64).

### 2.4. Molecular Detection of *Bartonella* Spp.

DNA was extracted from whole blood in EDTA tubes via a QIAamp DNA Blood Mini Kit (Qiagen, Germany) according to the manufacturer's instructions. DNA concentrations were assessed via a spectrometer. The A260/A280 ratios of all the samples obtained fell within the standard range of 1.7–1.9. To detect *Bartonella* spp., a series of nested PCR processes were executed, which employed a particular set of primers that were designed to focus on the 16S–23S rRNA ITS region [31]. Nested PCR was performed via AmpliTaq Gold 360 Master Mix (Thermo Fisher Scientific, USA) following the manufacturer's instructions. Primary PCR (target amplicon size: 753 bp) was performed using the following thermal cycling conditions: initial pre-denaturation at 95°C for 5 min, followed by five cycles of denaturation at 95°C for 30 s, annealing at 48°C for 30 s, and elongation at 72°C for 1 min, followed by 30 cycles of denaturation at 95°C for 30 s, annealing at 50°C for 30 s, and elongation at 72°C for 1 min. A final extension was performed at 72°C for 5 min. For nested PCR (target amplicon size: 599 bp), the thermal cycling conditions included initial denaturation at 95°C for 5 min, followed by 30 cycles of denaturation at 95°C for 30 s, annealing at 41°C–45°C for 30 s, and elongation at 72°C for minute. A final elongation step was conducted at 72°C for 5 min. Primary PCR was performed with the ITS_OF(5′- TTCAGATGATGATCCCAAGC-3′) and the ITS_OR(5′- AACATGTCTGAATATATCTTC-3′) primers, which amplified *B. henselae* (753 bp) fragments. Nested PCR amplified *B. henselae* (599 bp) with the ITS_IF(5′- CCGGAGGGCTTGTAGCTCAG-3′) and the ITS_IR(5′- CACAATTTCAATAGAAC-3′) primers. The specific primer information and PCR conditions are provided in Table 1.

### 2.5. Direct Sequencing and Phylogenetic Analysis

The products obtained from the nested PCR process were purified via a commercial kit (QIAquick PCR Purification Kit; Qiagen, Germany). Direct sequencing was performed via forward and reverse primers specific to the ITS gene (Table 1), and the nucleotide sequences were analyzed. The genotypes were subsequently confirmed via NCBI BLAST searches on the basis of the nucleotide sequences. Phylogenetic analysis was carried out via MEGA-X software [32].

### 2.6. Data Analysis

The questionnaire data for the study were collected on paper forms and entered into a Microsoft Access database. Data entry was validated by comparing the electronic records with the information on the forms. Statistical data were analyzed via commercially available software (IBM SPSS Statistics, version 26; IBM Co., USA). Group comparisons were conducted to confirm the odds ratios via the chi-square test for categorical variables. If the percentage of categories exceeded 20% with an expected frequency of <5, Fisher's exact test was performed alternatively. Statistical significance was set at *p* < 0.05 for all analyses.

## 3. Results

### 3.1. Samples (Human and Cats)

The samples were collected from a total of 35 animal hospitals, comprising 300 individuals and 126 cats. All the human participants were working in veterinary hospitals. They were categorized as follows: 128 were veterinarians, 148 were technicians, 18 were hospital managers, and six were pet groomers. Among the 300 workers, 126 also participated as cat owners in the study. Signalments for the human participants and cats are summarized in Table 2. All samples that were inappropriate for analysis due to issues in the storage and transportation process, as well as inadequate sampling, were excluded. Consequently, for humans, two EDTA whole blood samples, six serum samples, and two saliva samples were excluded from the analysis. For cats, seven EDTA whole blood samples, 18 serum samples, three saliva samples, seven nail samples, and 14 fecal samples were excluded from the analysis.

### 3.2. Serological Prevalence of *Bartonella* Spp.

The results of IFA analysis performed on a total of 294 human serum samples revealed a *B. henselae* IgG seroprevalence rate of 64.6% (190/294). In contrast, all 294 samples tested negative for *B. henselae* IgM, resulting in a seroprevalence rate of 0% (0/294). In companion cats, IFA analysis revealed a *B. henselae* IgG seroprevalence rate of 5.5% (6/108).

### 3.3. Molecular Prevalence of *Bartonella* Spp.

A total of 298 human whole blood samples were subjected to ITS nested PCR, resulting in a PCR positivity rate of 3% (9/298). In saliva samples, a positivity rate of 1.7% (5/298) was observed. For cats, nested PCR was performed on 119 whole blood samples, revealing a positivity rate of 10.1% (12/119). However, all 123 saliva samples from cats tested negative, resulting in a molecular seroprevalence rate of 0% (0/123). Additionally, nested PCR was conducted on nail and fecal samples from cats, with positivity rates of 1.7% (2/119) and 4.5% (5/112), respectively.

### 3.4. Phylogenetic Analysis of *Bartonella* Spp.

**Figure 1:**
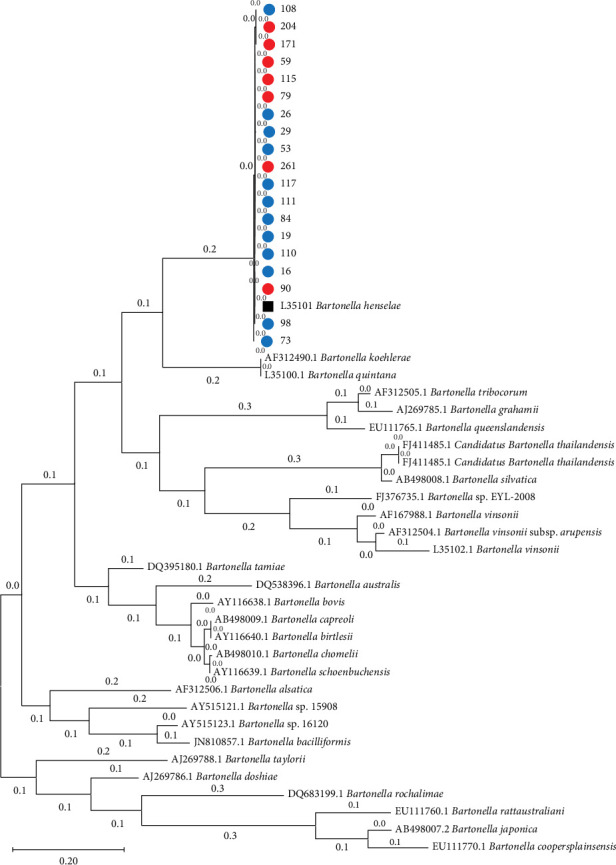
Phylogenetic tree based on ITS region sequences from *Bartonella* spp. isolates. Sequences obtained from human samples are shown in red, and those from animal (cat) samples are shown in blue.

For the PCR-positive blood samples subjected to nested PCR, direct sequencing was performed to confirm the species of *Bartonella* spp. isolated. The nested PCR targeting the ITS region (16S–23S rRNA intergenic spacer) of *B. henselae* yielded a 599 bp product, which was used for sequencing and phylogenetic analysis. The results indicated that all strains isolated from both human- and cat-positive samples were *B. henselae*. Consequently, the phylogenetic tree generated is presented in Figure 1.

### 3.5. Analysis of the Correlation Between Serological and Molecular Positive Samples

#### 3.5.1. Serological-Positive Human Samples

Among the 190 participants who tested serologically positive, 66 were identified as cat owners. Among these 66 cat owners, the seroprevalence of their companion cats was 4.5% (3/66). This corresponds to 50% (3/6) of the seropositive cats in this study. Among the 66 seropositive cat owners, five people also tested positive in blood PCR. The cats of three owners did not participate in this study, and other cats of two owners who participated in this study tested negative for both serum and PCR. These results are summarized in Table 3.

#### 3.5.2. Serological-Positive Cat Samples

For the six serologically positive cats, all samples (blood, saliva, nails, and feces) tested negative according to the PCR results. All three owners of the six seropositive cats were serologically positive; however, their blood and saliva PCR results were negative. These results are summarized in Table 4.

#### 3.5.3. Molecular-Positive Human Samples

Among the nine veterinary personnel who tested positive for *B. henselae* DNA in their blood, two were cat owners. However, the cats owned by two individuals tested negative in both the serological and PCR tests.

#### 3.5.4. Molecular-Positive Cat Samples

For the 12 cats that tested molecular-positive in blood, all of their owners tested negative in PCR results.

### 3.6. Questionnaire-Based Analysis

#### 3.6.1. Humans

Out of a total of 300 participants, 262 veterinary personnel responded to the questionnaire. Among them, 172 tested positive serologically, whereas 90 tested negative serologically for *Bartonella* spp. The results of an epidemiological survey related to the transmission of *Bartonella* spp. are summarized in Table 5.

The history of clinical signs commonly associated with CSD did not significantly differ between the seropositive and seronegative groups. Additionally, there was no correlation between a history of wounds and seropositivity.

The survey related to the transmission routes of *Bartonella* spp. included questionnaires about exposure to arthropods, overseas travel within the past 3 years, and a history of scratches from cats. In all of these, no significant differences were observed in terms of seropositivity.

Questions were asked regarding the exposure time to cats, which are known as major transmission routes for *Bartonella* spp. First, the correlation between daily working hours in veterinary hospitals and seropositivity was examined. While the difference was not statistically significant, it was observed that individuals who tested seropositive had a slightly greater proportion of individuals working at the hospital for more than 12 h a day (13.4% vs. 8.9%). Second, the cat owners were asked how much time they spend with their cats each day. Similarly, there was no statistical significance, but the number of cat owners spending more than 12 h a day with their cats was greater in the seropositive group (8.6% vs. 1.8%).

#### 3.6.2. Cats

Among the 126 cat owners included in the study, 108 samples could be analyzed. At the time of serum collection, no evidence of ectoparasite infestation was identified on physical examination in any of the cats. Among these, a survey was conducted on six cats that tested serologically positive. All seropositive cats were of the domestic shorthair breed. None of them had a recent history of hospitalization in veterinary clinics. The possibility of contact with stray cats was ruled out for all six cats, and only one of them had a history of scratching with other pet cats. All six cats were prescribed preventive medications for heartworms and internal parasites, but only three of them were also taking expellants against ectoparasites such as ticks. Moreover, only one cat received regular monthly preventive treatments.

## 4. Discussion

This study confirmed the prevalence of *Bartonella* infection among veterinary personnel in South Korea through serological and molecular methods. Additionally, it assessed the serological and molecular prevalence and transmission potential of *Bartonella* spp. among cat owners and companion cats. To screen for the risk of zoonotic transmission by companion cats, this study focused on veterinary workers with high cat exposure. In South Korea, the seroprevalence of *Bartonella* spp. in veterinary workers was significantly high, and molecular evidence of infection was observed even in humans without clinical signs related to CSD. In companion cats, the seroprevalence was not comparable with that reported previously, but the molecular prevalence could [28]. The PCR prevalence rates of companion cats in South Korea were much lower than those reported in a study conducted in 2009 (blood 10.1% vs. 33.3%; saliva 0% vs. 43.5%; and nail 1.7% vs. 29.5%). However, the cats in this study were owned by veterinary personnel and were likely to have received more consistent and comprehensive veterinary care, which may have influenced the findings. While evidence of direct transmission by cats was not confirmed through the analysis of prevalence, the potential for transmission through companion cats was still evident through questionnaires. The possibility of transmission from overseas and other vectors except for fleas was expected to be low on the basis of the questionnaire. In conclusion, this study indicates that *Bartonella* spp. is already widespread as a significant zoonotic pathogen in South Korea. Furthermore, there is a need for increased awareness of bartonellosis among veterinary workers considering the potential for transmission by cats.

In this study, the seroprevalence of *Bartonella* infection among veterinary personnel was high compared with that of healthy adults in a previous study (64.6% vs. 15.0%) [26]. The results suggest that people with high exposure to dogs and cats, such as veterinary workers, have a high rate of asymptomatic infection. This implies that companion animals may serve as a source of *Bartonella* transmission. In this study, we focused on people with the highest level of cat exposure to assess the potential for transmission, but further research through a comparison of prevalence between those who have little exposure to cats and veterinary workers is needed.

The seroprevalence of companion cats (5.5%) was much lower than that reported in Japan (9.1%), Italy (43.5%), Angola (66%), Spain (80.7%), and the USA (81%) in previous studies [33–37]. It is thought that differences in the indoor environment where companion cats live may have contributed to the lower seroprevalence than that reported overseas. In South Korea, shoes are not used indoors, and carpets are not used often; thus, it is more difficult for fleas to live in these environments than abroad. In the same context, the high rate of people living in apartments can also contribute. Furthermore, it could be affected by the fact that most companion cats in South Korea receive parasite prevention on a regular basis.

Although the difference was not statistically significant, the risk of being scratched by cats was approximately 1.9 times higher in serologically positive people than in negative people. Furthermore, in a question for only cat owners, the risk of all-day (>12 h) cases was 5.2 times higher in seropositive than seronegative people at the question of time spent with cats in the same space. Of course, there is a possibility that it occurred as a coincidence, but these results are consistent with the fact that cats are the main source of *bartonella* transmission to humans.

Generally, cats serve as major carriers for the transmission of *Bartonella* spp., as suggested by the name “CSD.” However, recently, there has been a possibility of direct transmission through arthropods. Owing to global warming, it is anticipated that there will be an increase in the number of arthropods in countries capable of transmitting *Bartonella* spp. different from those in the past. In fact, numerous studies have confirmed that the prevalence is higher in regions with a warmer climate [8, 38, 39]. According to our survey results, the possibility of direct transmission through these vectors is low in South Korea. However, according to research on the prevalence of *Bartonella* spp. in these vectors (ticks and mites), a 5.2%–19.1% molecular prevalence has still showed the potential of transmission in South Korea [40]. Therefore, further studies should be conducted to clarify direct transmission by arthropods.

All six seropositive cats were confirmed to be domestic shorthairs, which make up the majority of the cat samples. This result does not suggest the presence of specific high-prevalence breeds but rather clearly demonstrates that *Bartonella* spp. are already distributed among domestic breeds in South Korea that were not recently introduced from overseas. Additionally, based on the questionnaire results, the likelihood of *Bartonella* transmission from stray cats to seropositive companion cats appeared to be low. Given the potential for arthropod-borne transmission, only one of the six seropositive cats in this study had a complete history of regular ectoparasite prevention. This underscores the potential necessity of implementing routine ectoparasite control measures in companion cats, irrespective of seasonal variation or outdoor exposure.

In addition, all seropositive cats received appropriate heartworm prevention. However, only one cat was confirmed to receive these preventive agents regularly. Typically, several heartworm prevention medications are known to control fleas, but they do not cover other external parasites, such as ticks and lice. In addition, because most companion cats in South Korea are not taken for regular outdoor walks, there is a tendency to administer preventive medications for heartworms and external parasites less regularly in cats than in dogs. In this study, the history of parasite prevention in seropositive cats suggests that it might be essential to implement routine measures against both heartworms and external parasites to prevent zoonotic diseases.

In humans, PCR positivity was detected in 9 of 298 blood samples (3%), comparable to the 2.25% (2/89) reported among Spanish veterinarians in 2017 [41]. All PCR-positive individuals in this study were asymptomatic, aligning with previous report suggesting no clear association between PCR detection and clinical signs [41]. However, higher PCR prevalence has been observed in high-risk populations [42, 43], and *Bartonella* spp., including *B. henselae*, are known to cause chronic intravascular infections in apparently healthy individuals [1]. Furthermore, reactivation of latent infection has also been reported in immunocompromised or co-infected hosts [44]. Therefore, the PCR positivity observed in this study should not be interpreted as evidence that these individuals are entirely free from the risk of bartonellosis.

The molecular prevalence of cats' blood samples showed 10.1%, which is higher than those reported in Sweden (2.2%), Portugal (6.7%), the Czech Republic (8%), Turkey (8.2%), and Egypt (8%) [45–49]. However, it is markedly lower than the 33.3% PCR prevalence of pet cats reported in South Korea in 2009 [28]. Moreover, while the previous study identified PCR positivity rates of 43.5% in saliva and 29.5% in nails [28], this study demonstrated significantly lower rates of 0% and 1.7%, respectively. Notably, a subsequent study by Maggi et al. highlighted potential limitations in the specificity of the PCR primers targeting the 16S–23S intergenic spacer region used in earlier studies, as the primers developed by Jensen et al. [50] were shown to also amplify Mesorhizobium DNA [51]. This cross-reactivity may have led to an overestimation of Bartonella prevalence in the 2009 study, which could partially account for the difference between that study and ours. Furthermore, it is worth noting that while the previous study was confined to samples from the Seoul metropolitan area, our study encompassed a nationwide sampling, providing a more comprehensive overview of the current status of *Bartonella* spp. in South Korea. In the meanwhile, the observed reduction in molecular prevalence may be attributable to improvements in pet care and an increase in the population of dewormed cats over time.

In this study, the seroprevalence of cats determined by IFA was found to be low, even lower than the PCR prevalence in feline blood samples (10.1% vs. 5.5%). Such discrepancies between serological and PCR results are well documented in Bartonella infections, both in humans and animals. In particular, several studies have confirmed a lack of correlation between antibody titers and PCR positivity in humans [52, 53] and in dogs with Bartonella-associated hemangiosarcoma [54]. The low seroprevalence may reflect the actual serostatus of the cats or indicate seronegativity at the time of sampling, due to an inadequate interval after infection for the development of a diagnostically detectable antibody response. Additionally, there have been several reports that seroconversion does not always occur in *Bartonella* infections [55, 56]. Similar studies have also highlighted the variability in antibody production against *Bartonella*, suggesting that serological diagnostics may be insensitive [57–59]. Therefore, in interpreting the differences between serological and molecular prevalence of this study, it is crucial to consider the pathogen-specific characteristics of *Bartonella* spp.

This study has several limitations. First, the culture test, known as the gold standard for diagnosing Bartonella, did not performed in this study. As the culture of *Bartorella* spp. is extremely challenging due to stringent environmental conditions, there are only limited reported instances of success [60]. Further research is needed to confirm these findings at the level of culture in South Korea as well. Furthermore, obtaining bacterial strains through culture could establish a foundation for future studies on zoonotic diseases. Second, the IFA and PCR assays in this study were conducted to assess the serological and molecular prevalence in clinically healthy companion cats and high-risk veterinary personnel in South Korea. As these methods could not demonstrate direct transmission between humans and cats, the study instead identified potential risk factors for transmission through epidemiological analysis based on questionnaire responses. In the future, further studies should involve the isolation and confirmation of bacteria from owners and companion cats, who are regarded as suspected cases of direct transmission through a larger sample size.

This study is noteworthy for focusing exclusively on high-risk occupational groups and confirming both seroprevalence and molecular prevalence of *Bartonella* spp. in both humans and companion cats. Furthermore, this is the first study in South Korea to confirm the potential transmission of Bartonella from companion cats to humans. According to the results, in South Korea, *Bartonella* spp. has a relatively high prevalence even in healthy adults, underscoring the importance of heightened awareness regarding this zoonotic disease. In particular, enhanced preventive measures such as hygiene education, the use of personal protective equipment, and ectoparasite control are warranted for high-risk professionals, including veterinarians and technicians, who are frequently in close contact with cats to prevent the transmission of *B. henselae*.

## Figures and Tables

**Table 1:** ITS nested PCR primers and conditions for the molecular detection of *Bartonella* spp.

Primer name	Nucleotide sequences (5′- 3′)		Size (bp)
ITS_OF	TTCAGATGATGATCCCAAGC		753
ITS_OR	AACATGTCTGAATATATCTTC		

ITS_IF	CCGGAGGGCTTGTAGCTCAG		599
ITS_IR	CACAATTTCAATAGAAC		

**1^st^ PCR**	**Temperature**	**Time**	**Cycles**
**(639 bp)**			

Pre-denaturation	95°C	5 min	1 cycle

Denaturation	95°C	30 s	5 cycles
Annealing	48°C	30 s	
Elongation	72°C	1 min	

Denaturation	95°C	30 s	30 cycles
Annealing	50°C	30 s	
Elongation	72°C	1 min	

Post-elongation	72°C	5 min	1 cycle

**2^nd^ PCR**	**Temperature**	**Time**	**Cycles**
**(499 bp)**			

Pre-denaturation	95°C	5 min	1 cycle

Denaturation	95°C	30 s	30 cycles
Annealing	41°C–45°C	30 s	
Elongation	72°C	1 min	

Post-elongation	72°C	5 min	1 cycle

**Table 2:** Signalments for the human participants and cats.

Human participants (*n* = 300)		
Category	Categorization	Number
Age (year)	10–20	134 (44.7%)
	30–40	157 (52.3%)
	50–60	9 (3%)
	Total	300 (100%)

Sex	Men	96 (32%)
	Women	204 (68%)
	Total	300 (100%)

Job	Vet	128 (42.7%)
	Technician	148 (49.3%)
	Hospital manager	18 (6%)
	Pet groomer	6 (2%)
	Total	300 (100%)

Cats (*n* = 126)		
Category	Mean	
Age (year)	6.29 ± 4.25	

	**Categorization**	**Number**

Sex	Male	3 (2.4%)
	Female	6 (4.8%)
	Male neutered	61 (48.4%)
	Female spayed	56 (44.4%)
	Total	126

Breed	Domestic shorthair	70
	Persian	16
	Siamese	10
	Russian blue	7
	Turkish angora	4
	Scottish fold	3
	Minuet	2
	Ragdoll	2
	Selkirk rex	2
	Bengal	2
	Abyssinian	2
	Korat	1
	American shorthair	1
	American curl	1
	Munchkin	1
	Norway forest	1
	British shorthair	1
	Total	126

**Table 3:** Correlative analysis with both serological and molecular positive human samples.

Number	Human number	IFA	PCR (blood)	Cat number	Cat IFA	Cat PCR (blood)
1	0313B1111	Positive	Positive	Not participate in		
2	0327B1105	Positive	Positive	0327A2105	Negative	Negative
3	0330B1104	Positive	Positive	Not participate in		
4	0311B1102	Positive	Positive	Not participate in		
5	0104B1105	Positive	Positive	0104A2105	Negative	Negative

**Table 4:** Correlation analysis with seropositive cat samples.

Number	Cat number	Cat IFA	Cat PCR (blood)	Human number	Human IFA	Human PCR (blood)
1	0102A2102	Positive	Negative	0102B1102	Negative	Negative
2	0316A2104	Positive	Negative	0316B1104	Positive	Negative
3	0331A2107	Positive	Negative	0331B1107	Negative	Negative
4	0104A2101	Positive	Negative	0104B1101	Positive	Negative
5	0102A2101	Positive	Negative	0102B1101	Negative	Negative
6	0319A2105	Positive	Negative	0319B1105	Positive	Negative

**Table 5:** The results of the questionnaire associated with the transmission of *Bartonella* spp.

Category		Categorization	Serological positive	Serological negative	*p*-Value	Odds ratio
History of clinical signs associated with CSD	History	Experienced	92 (53.5%)	44 (48.9%)	0.479	1.202
		Nonexperienced	80 (46.5%)	46 (51.1%)		
		Total	172	90	—	—
	Clinical signs	Ocular disorders	9 (9.8%)	3 (6.8%)	0.751	1.482
		Mental/neurological disorders	23 (25%)	16 (36.4%)	0.170	0.583
		Scratch/wound	62 (67.4%)	27 (61.4%)	0.489	1.301
		Cardiopulmonary disorders	3 (3.3%)	2 (4.5%)	0.658	0.708
		Gastrointestinal disorders	22 (23.9%)	8 (18.2%)	0.451	1.414
		Total	92	44	—	—

Exposure of arthropods		Yes	28 (16.3%)	13 (14.4%)	0.698	1.152
		No	144 (83.7%)	77 (85.6%)		
		Total	172	90	—	—

Overseas travel within the last 3 years		Yes	44 (25.6%)	27 (30.0%)	0.445	0.8021
		No	128 (74.4%)	63 (70.0%)		
		Total	172	90		—

History of scratch from cats		Yes	21 (12.2%)	6 (6.7%)	0.161	1.947
		No	151(87.8%)	84 (93.3%)		
		Total	172	90	—	—

Daily exposure time with cats in the vet hospital		<6 h	45 (26.2%)	21 (23.3%)	0.616	1.164
		6–12 h	104 (60.4%)	61 (67.7%)	0.244	0.727
		>12 h	23 (13.4%)	8 (8.9%)	0.286	1.582
		Total	172	90	—	—

Daily time with cats in the same room (only cat owners)		Not marked	2	5	—	—
		<4 h	25 (35.7%)	14 (25.0%)	0.196	1.667
		4–8 h	22 (31.4%)	17 (30.4%)	0.897	1.051
		8–12 h	15 (21.4%)	19 (33.9%)	0.116	0.531
		Always (>12 h)	6 (8.6%)	1 (1.8%)	0.131	5.169
		Total	70	56	—	—

## Data Availability

The data that support the findings of this study are available from the corresponding author upon reasonable request.

## Supporting Information

Supporting Information Supporting Information file 1 presents the complete questionnaire titled *Animal Hospital Staff* Survey including the epidemiological information associated with bartonellosis.
