# Supplementary material for: Exploring the Zoonotic Risk of Bartonella henselae: A Serological and Molecular Investigation of Veterinary Personnel and Companion Cats in South Korea
Source: Transbound Emerg Dis. 2025 Oct 24;2025:2468636. doi: 10.1155/tbed/2468636 (PMC12578557; doi:10.1155/tbed/2468636)
Supplement: Supporting Information — file 1 presents the complete questionnaire titled Animal Hospital Staff Survey including the epidemiological information associated with bartonellosis. [file 2468636.f1.docx]

**Supplementary file 1.** Animal Hospital Staff Survey (To be completed by Animal Hospital Staff)

| **Staff name** |  | **Hospital** |  | **City** |  |
| --- | --- | --- | --- | --- | --- |
| **E-mail address** |  | | **Occupation** |  | |

| **Staff Questions** |
| --- |
| **※ Please read the questions below and mark (√) accurately (Questions 1-6).**  1. What is your gender?  ① Male  ② Female  2. What is your age group?  ① 10s-20s  ② 30s-40s  ③ 50s-60s  ④ 70s or older  3. Have you traveled, been on a business trip, or resided overseas?  ① Yes  ② No  -------------------------------------------------------------------------------------------------  If you answered (Yes) to question 3, please provide the following information:  3-1. If you have a history of entering or leaving the country, please list all the countries, regions, years, and periods of residence.  ① Country Name: _________ Region Name: __________ Year: __________ Period: ___________  ② Country Name: _________ Region Name: __________ Year: __________ Period: ___________ ③ Country Name: _________ Region Name: __________ Year: __________ Period: ___________ ④ Country Name: _________ Region Name: __________ Year: __________ Period: ___________ ⑤ Country Name: _________ Region Name: __________ Year: __________ Period: ___________  4. Please check if you have a history of visiting a hospital for any of the following symptoms, which are commonly associated with cat-scratch disease (CSD).  (Multiple checks possible)  ① Uveitis ② Fever ③ Lymphadenitis ④ Bite/scratch by a pet  ⑤ Bite/scratch by a stray animal ⑥ Insomnia ⑦ Fatigue ⑧ Memory impairment  ⑨ Drowsiness ⑨ Headache ⑩ Anxiety ⑩ Depression ⑫ Tremors ⑬ Vision impairment ⑭ Eye pain ⑭ Balance disorder ⑯ Urination disorder ⑰ Tachypnea ⑱ Tachycardia  ⑲ Chronic diarrhea ⑳ Weight gain ㉑ Loss of appetite    5. Please check if you have a history of being bitten by or exposed to the following arthropods. (Multiple checks possible)  ① Fleas ② Ticks ③ Lice ④ Bedbugs  6. Please check if you have a history of being bitten or scratched by the following animals.  (Multiple checks possible)  ① Pet dog ② Pet cat ③ Stray dog ④ Stray cat  **※ The following questions are for animal hospital staff. Please respond (Questions 7-8).**  7. What is the size of the animal hospital where you work?  ① Veterinary clinics (less than 6 staff)  ② Referral veterinary hospital (6 or more staff)  8. On average, how much time per day do you spend with cats at your workplace?  ① <6 hours ② 6-12 hours ③ More than 12 hours (all day) |

| **Owner Questions** |
| --- |
| **※ The following questions are about the owner and the cat (Questions 9-11).**  9. How long have you been together with your cat?  ① 6 months or less ② 6 months - 1 year ③ 1 year - 3 years  ④ 3 years - 6 years ⑤ 6 years - 9 years ⑥ 10 years or more  10. On average, how many hours per day do you spend in the same room as your cat?  ① <4 hour ② 4-8 hours ③ 8-12 hours ④ 12 hours or more (all day)  11. On average, how many hours per day do you have direct contact with your cat? (Holding and playing with your cat)  ① Within 30 minutes ② 30-60 minutes ③ 1-2 hours ④ More than 2 hours |
